# Power Analysis for Human Melatonin Suppression Experiments

**DOI:** 10.3390/clockssleep6010009

**Published:** 2024-02-26

**Authors:** Manuel Spitschan, Parisa Vidafar, Sean W. Cain, Andrew J. K. Phillips, Ben C. Lambert

**Affiliations:** 1 Department of Health and Sport Sciences, TUM School of Medicine and Health, Technical University of Munich, 80992 Munich, Germany; 2 TUM Institute for Advanced Study (TUM-IAS), Technical University of Munich, 85748 Garching, Germany; 3 Max Planck Research Group Translational Sensory and Circadian Neuroscience, Max Planck Institute for Biological Cybernetics, 72076 Tübingen, Germany; 4 Faculty of Medicine and Health, Central Clinical School, University of Sydney, Sydney, NSW 2006, Australia; parisa.vidafar@sydney.edu.au; 5 Turner Institute for Brain and Mental Health, School of Psychological Sciences, Monash University, Clayton, VIC 3800, Australia; sean.cain@monash.edu (S.W.C.); andrew.phillips@monash.edu (A.J.K.P.); 6 Department of Statistics, University of Oxford, Oxford OX1 3LB, UK

**Keywords:** melatonin suppression, non-visual effects of ligh, statistical analysis, power analysis, experimental design

## Abstract

In humans, the nocturnal secretion of melatonin by the pineal gland is suppressed by ocular exposure to light. In the laboratory, melatonin suppression is a biomarker for this neuroendocrine pathway. Recent work has found that individuals differ substantially in their melatonin-suppressive response to light, with the most sensitive individuals being up to 60 times more sensitive than the least sensitive individuals. Planning experiments with melatonin suppression as an outcome needs to incorporate these individual differences, particularly in common resource-limited scenarios where running within-subjects studies at multiple light levels is costly and resource-intensive and may not be feasible with respect to participant compliance. Here, we present a novel framework for virtual laboratory melatonin suppression experiments, incorporating a Bayesian statistical model. We provide a Shiny web app for power analyses that allows users to modify various experimental parameters (sample size, individual-level heterogeneity, statistical significance threshold, light levels), and simulate a systematic shift in sensitivity (e.g., due to a pharmacological or other intervention). Our framework helps experimenters to design compelling and robust studies, offering novel insights into the underlying biological variability in melatonin suppression relevant for practical applications.

## 1. Introduction

Light exposure has a profound impact on human physiology and behaviour. In addition to enabling vision, light elicits a range of physiological and behavioural responses including the acute suppression of nocturnal melatonin production by light and shifting of the endogenous circadian rhythm [1,2]. These effects, often summarized under the umbrella term ‘non-visual’ effects of light [3], are mediated by a pathway connecting the eye to the hypothalamus. More specifically, the suprachiasmatic nuclei (SCN) receive retinofugal input, predominantly from a subset of retinal ganglion cells which are photosensitive in the absence of input from the cones and rods, the canonical photoreceptors underlying visual function. This photosensitivity is owed to the expression of the short-wavelength-sensitive photopigment melanopsin [4,5,6,7]. There is converging and convincing evidence linking the spectral sensitivity of melanopsin to the in vivo sensitivity of circadian and neuroendocrine responses to light [8,9,10,11,12,13].

Most evidence for the melatonin-suppressive effects of light is generated in laboratory experiments, in which participants are exposed to carefully controlled illumination (for examples, see [14,15,16,17]), while their saliva, plasma, or urine is collected for later melatonin assay [18,19]. As these experiments can be resource-intensive in terms of participant burden, processing costs, and staff to run a specific multi-hour (and potentially multi-day) experimental protocols, an important consideration in designing experiments is statistical power.

Non-visual sensitivity to light exhibits large individual differences [20,21], as demonstrated by a recent study by Phillips et al.  [22], in which participants were exposed to overhead fluorescent illumination at different illuminance levels on different evenings (10 lx, 100 lx, 200 lx, 400 lx, 1000 lx, 2000 lx). During this time their melatonin secretion was measured, showed substantial individual differences, with the most sensitive individual being up to  60 times more sensitive than the least sensitive individual in their sample.

These sizeable individual differences require special attention in designing experiments to obtain compelling evidence, particularly when it is not feasible to run extensive within-subjects experiments sampling the same individuals multiple times under multiple illuminances (thereby minimising individual differences). Here, we present a novel framework for facilitating power analyses for human melatonin suppression experiments to optimize the choice of experimental illumination levels.

## 2. Results

### 2.1. Overview

**Figure 1:**
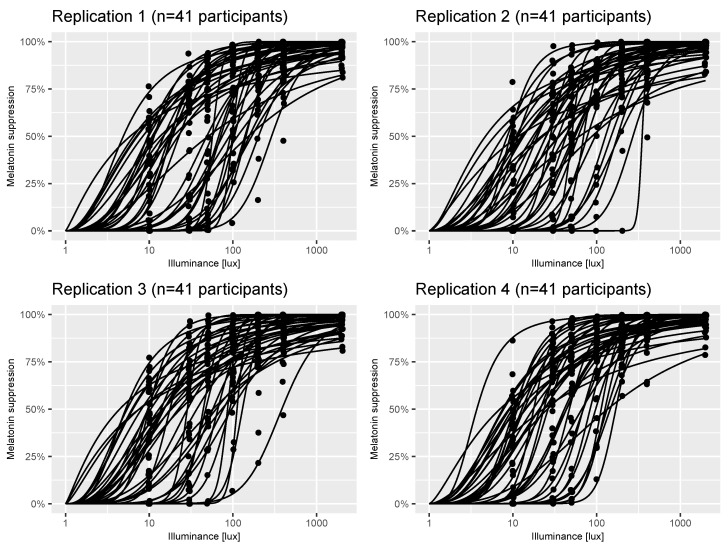
Example virtual experiments. Each panel shows individual dose-response curves and measurements (black points) of melatonin suppression at a series of illuminance values for *n* = 41 participants generated using the virtual_-_experiment (n = 41) function from the melluxdrc R package. The assumptions underpinning each of these panels are described in Section 2.2.

We developed a Bayesian statistical model of a virtual laboratory melatonin suppression experiment [22]. The model can be thought of as representing the melatonin suppression versus photopic illuminance [lx] curves (henceforth termed “dose-response curves”) across the population from which the original study participants were sampled. The model is stochastic, meaning that each virtual individual drawn from it will likely have a different dose-response curve. Figure 1 shows four replicates of a virtual experiment comprising n=41 participants in each case (the same sample size as the estimates in [22]). There are two types of stochasticity inherent in the model: individual-level variation in dose-response curve parameters; and measurement error when recording an individual’s melatonin suppression level at a single discrete illuminance value. These two sources of uncertainty are evident in Figure 1 by the variation in dose-response curves between subjects and the variation in measurements (black points) around them.

The assumptions underpinning our model of virtual experiments are detailed in Section 2.2. The ability to generate such virtual experiments allows us to conduct a series of *in silico* experiments to estimate the statistical power for a series of different laboratory study designs. The various study types are outlined in Section 2.3. As part of this work, we have created an open-source R package called *melluxdrc* [23] that wraps the functionality required to generate virtual experiments and to perform statistical power calculations. To facilitate power calculations for those designing experiments, we have also conducted a series of calculations and made those results freely available via an online R Shiny application called the *mellux-app* [24], which we describe briefly in Section 5.3.

### 2.2. A Model of Virtual Experiments

Our model of virtual experiments comprises two elements: (a) a population model of dose-response curves and (b) a measurement error model that statistically represents the various noise factors that influence measurements of melatonin suppression at a particular lux level.

The population model of dose-response curves is based on parameter estimates presented for n=41 participants in [22]. In [22], dose-response data for n=55 participants were obtained in a within-subjects protocol, where participants were exposed to a dim control (<1 lux) and five other experimental light levels (10, 30, 50, 100, 200, 400, 2000 lux) for 5 h in the evening. Using these data, melatonin suppression values, s(x,i), were obtained at each experimental light level, *x* for each participant *i*. The series of melatonin suppression values for each participant were then modelled using a logistic-type curve of the form:

(1) $$s\left(x,i\right)=1-\frac{1}{1+{\left(\frac{\log_{10}x}{a_{i}}\right)}^{b_{i}}},$$

which has the property that melatonin suppression increases towards 100% as illuminance increases without bound; here, $b_{i}>0$ controls the shape of each individual’s dose-response curve, and $a_{i}=\log_{10}{\mathrm{ED}}_{50}$ for that individual. Here, $ED_{50}$ is the dose corresponding to a melatonin suppression value of 50%.

The values used for our analysis were the $\left(a_{i},b_{i}\right)$ estimates for the n=41 participants previously reported in which a reliable estimate of the dose-response curve was obtained (less than 1 log-unit 95% confidence interval for the estimate of $a_{i}$): each set characterising the dose-response curve as in Equation (1). From here onwards, we refer to this set of estimates as the raw dose-response estimates. Using these values, we sought to create a statistical population model that represented these collection of dose-response curves which, crucially, could be sampled from to obtain a dose-response curve for a virtual individual.

**Figure 2:**
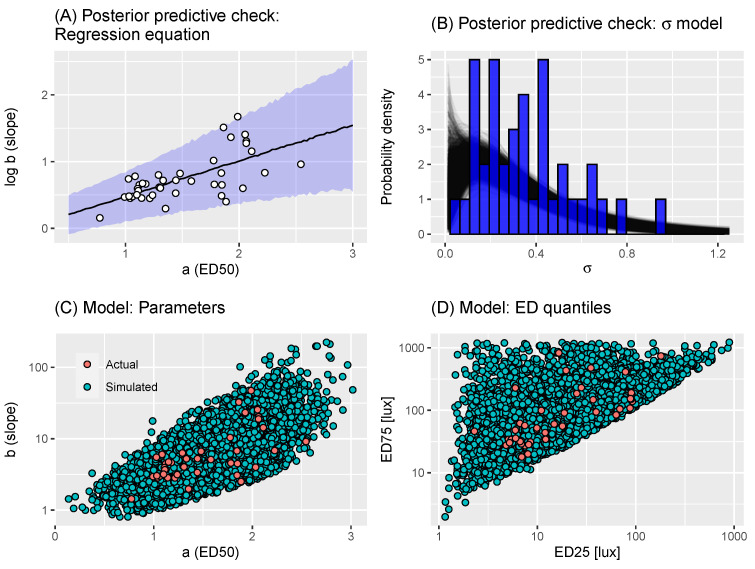
(**A**) Posterior predictive check: regression equation. Plot shows a graphical check of the model fit of Equation (2). The individual points show the estimates of $\left(a_{i},b_{i}\right)$ parameters provided by the authors of [22]. The uncertainty ribbon indicates the 2.5–97.5% posterior predictive quantiles; the black line indicates the 50% posterior predictive quantile. (**B**) Posterior predictive check: $\sigma_{i}$ model. Plot shows a graphical check of the model fit of Equation (4). Each black line represents a gamma density function corresponding to particular posterior samples of the parameters. The blue bars indicate the values of $\sigma_{i}$ estimated by the root-finding algorithm. (**C**) Assessing virtual individual generation: dose-response parameters. Each orange point represents a draw of (a,b) parameters (in Equation (1)) obtained via Algorithm 1: here, we show 25,000 such estimates; each green point represents raw estimates from [22]. (**D**) Assessing virtual individual generation: $ED_{x}$ quantiles. Each orange point represents the $\left(ED_{25},ED_{75}\right)$ values correspond to a draw of (a,b) parameters (in Equation (1)) obtained via Algorithm 1: here, we show 25,000 such estimates; each green point represents the $ED_{x}$ quantiles corresponding to the raw estimates from [22].

To do so, we built a statistical distribution representing the raw estimates. To represent this bivariate distribution, we used kernel density estimation using the bkde function from the KernSmooth R package [25] to approximate the empirical distribution over $a_{i}$ values. We then modelled the conditional distribution of $\log b_{i}$ conditional on $a_{i}$ through a regression equation:

(2) $$\log b_{i}∼\mathrm{normal}\left(\alpha +\beta a_{i},\sigma_{0}+\sigma_{1}a_{i}\right),$$

where the regression equation allows heteroscedasticity, represented by a standard deviation that increases linearly with $a_{i}$ (since $\sigma_{0}>0$ and $\sigma_{1}>0$). This model was estimated using a Bayesian framework, meaning that priors were set on the parameters. The priors chosen were uninformative, allowing a wide range of possible relationships and are shown in Table 1. The model was fitted using Markov chain Monte Carlo (MCMC) through Stan’s NUTS algorithm [26], using 4 Markov chains with 4000 iterations per chain, with 2000 of each chain’s iterations discarded as warm-up; finally, the post-warm-up iterations were thinned by a factor of 2. MCMC convergence was diagnosed through $\hat{R}<1.01$ and having bulk- and tail-ESS values above 400 [27]. Posterior predictive checks of model fit (see, for example, [28]) indicated that the model was a reasonable fit to the data (Figure 2). The Stan file and all materials needed to reproduce this analysis are available at [29].

**Figure 3:**
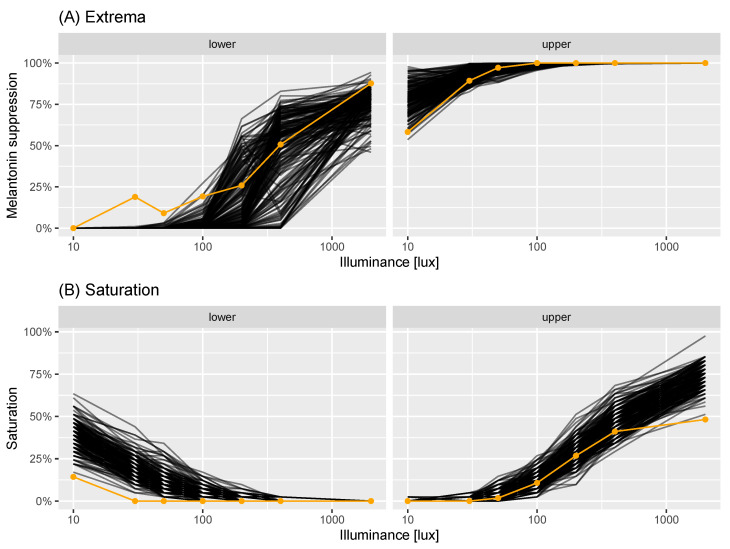
Virtual experiment check: saturations. Panels correspond to lower (percentage of observations <5%) and upper (percentage of observations >95%) saturations. Each black line corresponds to saturations generated from a single virtual experiment of sample size n=41. Each orange point corresponds to the real saturation.

To sample parameters characterising a dose-response curve for a virtual individual, we then used the approach described in Algorithm 1. Briefly, the first section of the algorithm draws *a* from the kernel density estimate of the empirical distribution. Then, Equation (2) is used to draw a value of logb conditional on *a*. The final section of the algorithm calculates the $ED_{25}$ and $ED_{75}$ corresponding to the sampled *a* and *b* values. If either of these are more extreme than thresholds derived from the corresponding ED values from the estimates from [22], those (a,b) values are rejected and the function is called again. This last step ensures that the dose-response curves obtained for virtual individuals are not far more extreme than those witnessed in the raw estimates. This approach is able to generate samples of (a,b) parameters that encompass the distribution of raw estimates (Figure 3). Accordingly, the corresponding $ED_{25}$ and $ED_{75}$ values generated by this process were also a reasonable fit to those corresponding to the raw estimates (Figure 3).
**Algorithm 1 Virtual individual generation.** Takes as input posterior draws of $\alpha ,\beta ,\sigma_{0},\sigma_{1}$ in Equation (2)1:**procedure** VirtualIndividual($\alpha ,\beta ,\sigma_{0},\sigma_{1}$)2:    Sample *a* from a kernel density approximation of empirical distribution using inverse transform sampling3:    Uniformly sample a set of random $\left(\alpha ,\beta ,\sigma_{0},\sigma_{1}\right)$ values from posterior draw set4:    Use Equation (2) to draw a value of logb (and hence *b*)5:    Use $ed\left(x\right)=10^{a{\left(-1+1/\left(1-x\right)\right)}^{1/b}}$ to calculate $ED_{25}$ (x=0.25) and $ED_{75}$ (x=0.75) for that individual6:    If $ED_{25}$ is less than half the minimum $ED_{25}$ in estimates from [22], reject (a,b) and call VirtualIndividual7:    If $ED_{75}$ is greater than 1.5 times the maximum $ED_{75}$ in estimates from [22], reject (a,b) and call VirtualIndividual8:    **return** (a,b) which characterise a dose-response curve9:**end procedure**

**Figure 4:**
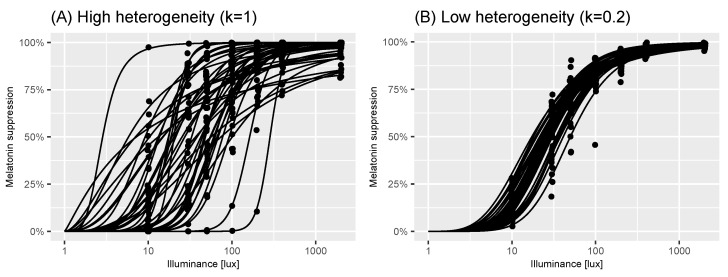
Effect of reduction in individual variance. Panel A shows individual dose-response curves and measurements (black points) of melatonin suppression at a series of illuminance values for n=41 participants generated using virtual_-_experiment (n = 41) function from the melluxdrc R package. Panel B shows the same but assuming a reduction in individual variance given by η=0.2 as given by virtual_-_experiment (n = 41, individual_-_variation_-_level = 0.2). The assumptions underpinning each of these panels are described in Section 5.

As part of our virtual population, we also developed an algorithm to allow users to simulate a reduction in individual heterogeneity in the population. To do so, we introduced a parameter, 0≤η≤1 that modulates the level of individual heterogeneity in the population: here, η=0 indicates that virtual individuals sampled from the population all have the same dose-response curve near the population median; η=1 indicates that the virtual individuals are drawn from the unrestricted population model (i.e., by the same process described in Algorithm 1). The process used to sample virtual individuals from the population is provided in Algorithm 2. Figure 4 shows how reducing the individual-level heterogeneity using this approach results in a tighter spread of dose-response curves.
**Algorithm 2 Virtual individual generation: reduced individual variance.** Takes as input posterior draws of $\alpha ,\beta ,\sigma_{0},\sigma_{1}$ in Equation (2) and η1:**procedure** VirtualIndividualReducedVariance ($\eta ,\alpha ,\beta ,\sigma_{0},\sigma_{1}$)2:    For all $\alpha_{i}$ posterior draws, shrink towards the grand mean: $\alpha_{i}=\alpha_{i}+\left(\bar{\alpha }-\alpha_{i}\right)\left(1-\eta \right)$ where $\bar{\alpha }$ is the posterior mean3:    For all $\beta_{i}$ posterior draws, shrink towards the grand mean: $\beta_{i}=\beta_{i}+\left(\bar{\beta }-\beta_{i}\right)\left(1-\eta \right)$ where $\bar{\beta }$ is the posterior mean4:    Uniformly sample a set of random (α,β) values from the variance-reduced posterior draw set5:    Uniformly sample a set of random $\left(\sigma_{0},\sigma_{1}\right)$ values from the posterior draw set6:    Sample $a^{\prime }$ from a kernel density approximation of empirical distribution using inverse transform sampling (see [30])7:    Calculate $a=a^{\prime }+\left(a_{50}-a^{\prime }\right)\left(1-\eta \right)$ where $a_{50}$ indicates the median value of the empirical distribution8:    Calculate $\sigma =\eta (\sigma_{0}+\sigma_{1}a)$9:    Draw a value of $\log b∼\mathrm{normal}(\alpha +\beta a_{i},\sigma )$10:    Use $ed\left(x\right)=10^{a{\left(-1+1/\left(1-x\right)\right)}^{1/b}}$ to calculate $ED_{25}$ (x=0.25) and $ED_{75}$ (x=0.75) for that individual11:    If $ED_{25}$ is less than half the minimum $ED_{25}$ in estimates from [22], reject (a,b) and call VirtualIndividualReducedVariance12:    If $ED_{75}$ is greater than 1.5 times the maximum $ED_{75}$ in estimates from [22], reject (a,b) and call VirtualIndividualReducedVariance13:    **return** (a,b) which characterize a dose-response curve14:**end procedure**

As is seen in Figure 2 in [22], the measured melatonin suppression values at discrete illuminance values exhibited often considerable variation around the best fit lines. Using the Root-Mean-Square-Errors (RMSEs) for each fit in for each individual, we developed an error model representing measurement variability for each individual. To do so, we assumed that measurement noise was additive on the logit scale to ensure that measurements could not fall outside of the [0, 1] range. That is,

(3) $$\mathrm{logit}\;\tilde{s}\left(x,i\right)∼\mathrm{normal}\left(\mathrm{logit}\;s\left(x,i\right),\sigma_{i}\right),$$

where logit(z):=logz/(1−z), and $\tilde{s}\left(x,i\right)$ represents the measured melatonin suppression at illuminance *x* for individual *i*. Here, $\sigma_{i}>0$ is an individual-specific value quantifying measurement noise.

We aimed to determine the value of $\sigma_{i}$ that generated measurement noise resulting in a corresponding RMSE value close to that observed for individual *i*. To do so, we used an approximate estimation approach where, in each iteration, we simulated data using Equations (1) and (3), and compared the simulated RMSE value to the truth. Specifically, we used the known (estimated) $\left(a_{i},b_{i}\right)$ values for individual *i* to simulate a dose-response curve given by Equation (1); we then generated measurements at the discrete illuminance values used in [22] using Equation (3) with a particular $\sigma_{i}$. For each such “experiment”, we calculated an RMSE value. For a given $\sigma_{i}$ value, we repeated the experiment 100 times, and calculated an average RMSE value across all replicates. The difference between this average simulated value and the true RMSE value was then used as the target for the one-dimensional root-finding algorithm uniroot available in R: the output of this algorithm was, hence, a value of $\sigma_{i}$ for each individual.

In order to generate measurements for virtual individuals, we needed an approach to generate potential $\sigma_{i}$ values for unseen individuals. To do so, we assumed that the individual $\sigma_{i}$ values were drawn from the following population process:

(4) $$\sigma_{i}∼\mathrm{gamma}\left(c,d\right),$$

where c>0 and d>0. We estimated the parameters in Equation (4) using the values of $\sigma_{i}$ estimated by the root-finding algorithm. The model was estimated in a Bayesian paradigm, requiring that we set priors on the parameters. Here, we chose uninformative priors on the parameters, which are specified in Table 1. The model was fitted using Markov chain Monte Carlo (MCMC) through Stan’s NUTS algorithm [26] using 4 Markov chains with 2000 iterations per chain, with 1000 of each chain’s iterations discarded as warm-up. The same criteria were used to diagnose model convergence as for Equation (2). Posterior predictive checks of model fit indicated that the model was a reasonable fit to the data (Figure 2).

With both elements of the virtual experiment model in place—the population model of dose-response experiments and the measurement error model—we sought to assess how well the resultant model represented the data collected in [22]. This was tested using the minimum and maximum melatonin suppression at each measured illuminance value (the “extrema”); and the percentage of observations which were extreme at each lux: either below 5% lux or above 95% melatonin suppression (the lower and upper “saturation values”). We generated 200 replicate virtual experiments, which each had the same number of individuals (n=41) as the estimates we were provided with. For each experiment and illuminance value, we calculated simulated extrema and saturation values to compare with the real measurements. In Figure 3, we compare the modelled extrema (black lines) with the real values (orange points and line): in the left-hand plot, we compare the minima; in the right plot, we compare the maxima. This indicates that, for moderate-high illuminance values, the modelled and real values were in good accordance. At the lower illuminance values, the modelled values tended to be more extreme (either closer to 0% for the minima; or closer to 100% for the maxima) than the real data. In Figure 3, we performed the same comparison but for the levels of simulated and actual saturations. In this case, the upper saturation values between the modelled and real data were reasonable, apart from at the highest illuminance value measured. The lower saturations exhibited the same pattern as for the extrema, in that the simulated data were more extreme than the actual.

The discrepancies between the modelled and actual data could be due to (a) assumptions around our model for the population of dose-response curves, (b) assumptions around our measurement process model and/or (c) issues with the original logistic models used to model dose-response curves in [22]. Our checks for our model of dose-response curves (Figure 3) indicate no issue with representing the original estimates. It is possible that the logit-normal noise process we assume in Equation (3) results in greater variation than seen in reality. Visual inspection of Figure 2 in Phillips et al. [22], however, shows that, for a number of individuals, the logistic dose-response curve is downward-biased for low illuminance levels, potentially indicating that a two-parameter logistic is inappropriate in this extreme. Without the raw experimental data, however, it is not possible to determine the exact cause of the discrepancy between modelled and real life data. Despite these differences, the overall correspondence between the model and data is good, and, as such, it provides a reasonable basis to determine statistical power for a variety of different experimental settings.

### 2.3. Power Calculations

**Figure 5:**
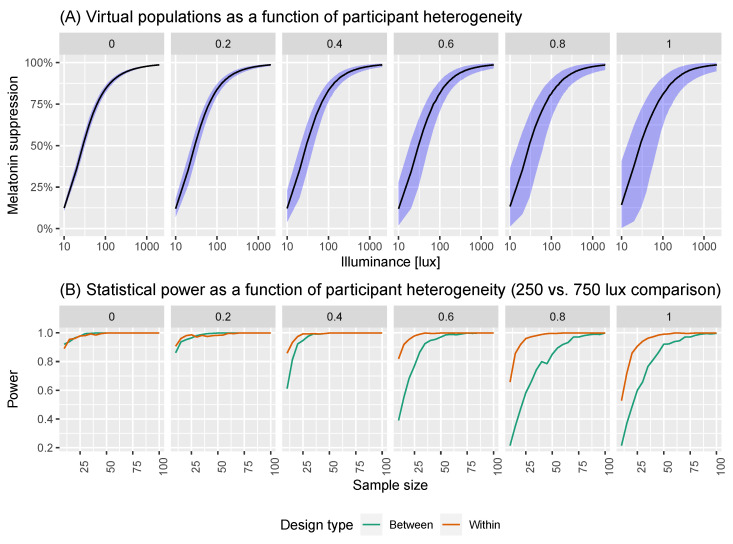
(**A**) Virtual populations as a function of participant heterogeneity. Here, we are simulating the group dose-response curves assuming different levels of participant heterogeneity. (**B**) Statistical power as a function of participant heterogeneity.

**Figure 6:**
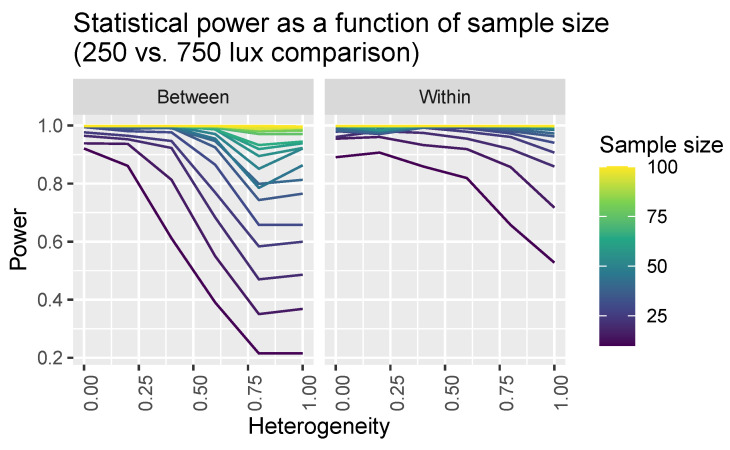
Statistical power for detecting a difference in melatonin suppression assuming different samples sizes and between-subjects (**left panel**) and within-subjects (**right panel**) designs.

Having developed a virtual experiment model (see Section 2.2), we sought to use it to help inform experimental design. In particular, we considered two classes of experiment: in illuminance comparison experiments, the aim is to quantify whether population melatonin suppression differs across two measured illuminance values; in intervention experiments, the aim is to estimate the effect size for an intervention that systematically changes the dose-response curves (i.e., changes light sensitivity). Within each experimental class, we considered two possible experimental designs: within-subject designs, where the same participants are measured twice; or between-subject designs, where two separate groups of participants are measured once each. In all cases, we used our model of virtual experiments to generate data that mimics the types of laboratory experiments described here, which was then used to determine statistical power(Figure 5 and Figure 6).

In two-level experiments, individual melatonin suppressions are measured at two photopic illuminance levels: $x_{1}$ and $x_{2}$. In within-subject experiments, each individual is measured twice resulting in *N* paired observations: $D={\left\{\tilde{s}\left(x_{1},i\right),\tilde{s}\left(x_{2},i\right)\right\}}^{1:N}$. In between-subject experiments, there are 2N individuals, with *N* measured at $x_{1}$ and another *N* at $x_{2}$, resulting in a dataset: $D=\{{\left\{\tilde{s}\left(x_{1},i\right)\right\}}^{1:N}$ & ${\left\{\tilde{s}\left(x_{2},i\right)\right\}}^{N+1:2N}\}$. In these experiments, the aim is to determine that there is a significant difference in the suppression response between measurements taken at $x_{1}$ and $x_{2}$. To do so, we use a *t*-test which is either paired (for within-subject designs) or independent (for between-subject designs). We considered all possible pairs of different illuminance values from the set {10, 30, 50, …, 1970, 1990}. For each pair, we also considered a range of sample sizes from N=10, 20, …, 90, 100 and a range of possible individual heterogeneity values: η=0, 0.2, 0.4, 0.6, 0.8, 1.0. For each combination of these parameters, we calculated the power to detect significant differences of the correct sign using test sizes of 1%, 5% and 10%.

**Figure 7:**
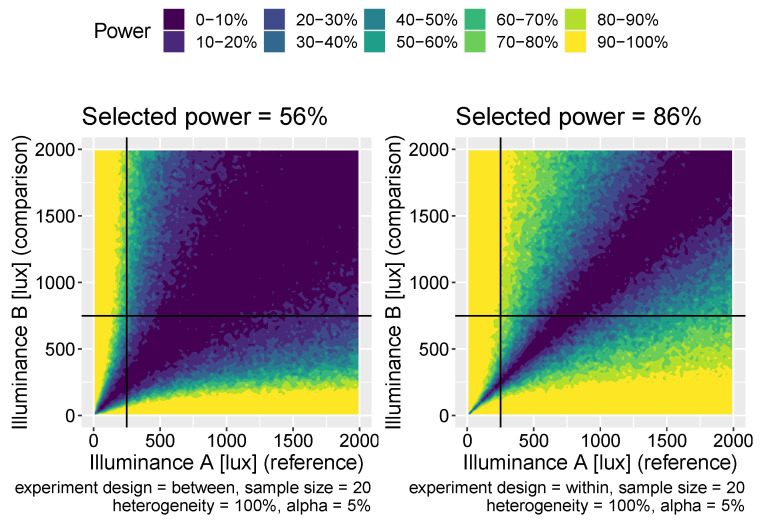
Power maps for two-illuminance comparison experiments for between-subjects (**left panel**) and within-subjects experiments (**right panel**). The choice of comparison light levels here is 250 and 750 lux.

We show visualisations of the outcomes for these analyses in Figure 6 and Figure 7. Figure 6 shows the statistical power as a function of participant level hetereogeneity for between- and within-subjects designs. The same principle of power but for different illuminances levels is shown in Figure 7. The results are clear: within-subjects designs beat between-subjects designs.

In intervention experiments, we assume interventions that shift the ${\mathrm{ED}}_{50}$ away from the baseline level. In within-subject study designs, individuals are assumed to have their melatonin suppressions measured at a particular illuminance level (in lux) *x* once before the intervention occurs and once afterwards, resulting in a dataset: $D={\left\{\tilde{s}\left(x,i\right),{\tilde{s}}^{\prime }\left(x,i\right)\right\}}^{1:N}$ where unprimed variables indicate natural levels and primed indicate intervention levels. For the between-subject design, two different sets of individuals were measured: a “baseline” group and an “intervention” group whose ${\mathrm{ED}}_{50}$ values are shifted. This resulted in a dataset $D=\{{\left\{\tilde{s}\left(x,i\right)\right\}}^{1:N}$ & ${\left\{{\tilde{s}}^{\prime }\left(x,i\right)\right\}}^{N+1:2N}\}$. As for the illuminance comparison experiments, we used *t*-tests to compare the natural and intervention melatonin suppressions: again using paired-tests for the within-subject design and independent tests for the between-subject design. We considered all illuminance values from the set x={10, 30, 50, …, 1970, 1990} across the same set of sample sizes and individual heterogeneities as for the comparison experiments. Additionally, we considered natural ${\mathrm{ED}}_{50}$ multipliers taking values χ={0.2, 0.4, …, 1.8, 2.0}, where, for example, χ=0.2 means the natural ${\mathrm{ED}}_{50}$ of individuals are reduced by a factor of 5. For each combination of these parameters, we calculated the power to detect significant differences of the correct sign using test sizes of 1%, 5% and 10%.

**Figure 8:**
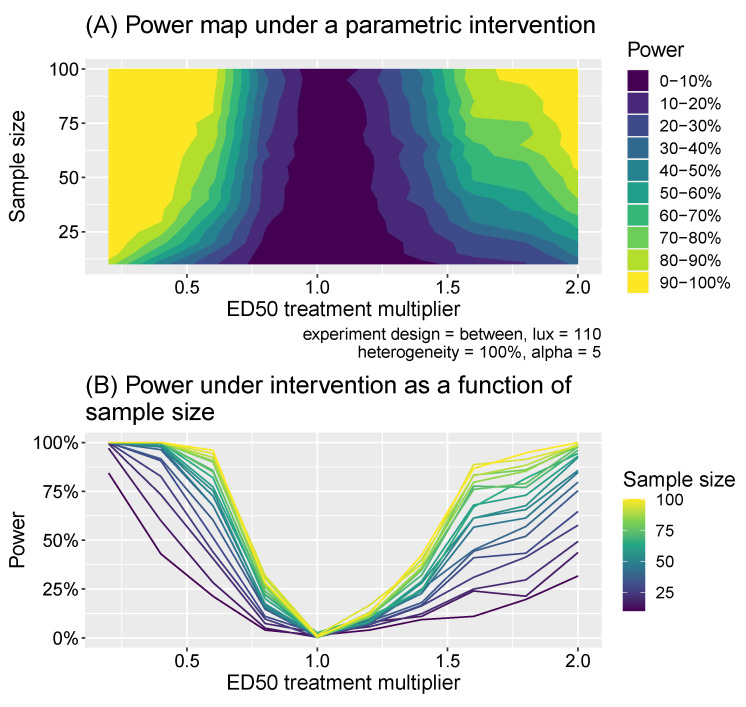
Panel (**A**) shows the power as a function of the intervention effect (expressed as a multiplier of the ED50 value) and sample size. Panel (**B**) shows a slice of the same data as a line plot for different discrete samples sizes.

We summarize the outcomes of these analyses in Figure 8. We find clear differences in power assuming a specific intervention effect. These analyses in Figure 8 may serve to inform sample size calculations for planning interventions affecting the impact of light on melatonin suppression.

### 2.4. Extension to Melanopic EDI

In the present model, we used photopic illuminance in lux as the independent variable. It is well known that melatonin suppression follows the spectral sensitvity of melanopsin [7,12]. In the experiments underlying our model [22], the spectrum did not change substantially with different intensities. As a consequence, the results here hold independent of the light quantification metrics, and using a simple scaling factor, can be expressed as melanopic equivalent daylight illuminance (mEDI) [31]. Following calculations with luox [32], the melanopic EDIs were 1.58 lx (nominal 10 photopic lx), 48.24 lx (nominal 100 photopic lx), 99.76 lx (nominal 200 photopic lx), 206.33 lx (nominal 400 photopic lx) and 1106.21 lx (nominal 200 photopic lx) in the original data set.

## 3. Discussion

### 3.1. Limitations

The presented model has notable limitations stemming from the reliance on a single data set, posing challenges regarding the generalizability of our findings. The modelling approach is flexible to allow for the future integration of data sets, should these become available (see Section 2.3). The dataset was collected in a relatively homogeneous group of participants. Indeed, given the large variability in even this rather homogeneous sample, it is unlikely that more heterogeneous samples would lead to less variable results. Future work include data from diverse populations.

Due to data-sharing restrictions, only individual-level logistic fits to the data were available. This limitation raises concerns about the assumed form of the dose-response curve and the appropriateness of the chosen noise model, both of which could be better assessed with access to raw individual-level data. Additionally, the assumed form of the noise model, specifically its additive nature on the logit scale, might not be the optimal fit.

The model exhibited strengths in providing a good fit to the available data, showcasing its reliability for the bulk of the available information. Furthermore, the approach emphasizes transparency and openness by making the model accessible through reproducible code repositories and a graphical user interface. The approach’s innovative aspect lies in being one of the initial attempts to construct a generative model of experimental data, allowing users to specify assumptions about individual-level variation. The use of a Bayesian approach is statistically principled by correctly incorporating various sources of uncertainty into the model’s characteristics, enhancing its robustness.

### 3.2. Future Directions

The analysis and approach presented here provides a novel approach for power analysis in human melatonin suppression experiments. It can help to inform future studies of melatonin suppression under various manipulations, assuming individual variability, and thereby support experimental design and a principled decision about samples sizes. Importantly, the framework presented here can be updated when more datasets, and more diverse data sets, collected under similar experimental paradigms become available. The approach proposed here for modelling dose-response curves can be extended to other dose-response relationships as well. An additional consideration is the inclusion of melanopic metrology into the modelling framework, along with more sophisticated photoreceptor integration models.

## 4. Conclusions

Here, we developed a model of virtual melatonin suppression experiments under different light levels informed by, and fitting to, actual experimental data. We expanded our model virtual experiments by allowing the modulation of individual-level variability and implementing a virtual intervention leading to a left- or rightward shift of the dose-response curve. We provide a web applet for exploring the virtual model and using it in practical applications.

## 5. Methods

### 5.1. Sample Characteristics

The study on which we base the statistical modeling is based on a sample of young healthy adults, aged 18–30 years (mean ± SD; age of 20.8 ± 2.6 y). Data from 41 participants were included in our analyses. The empirical sample had the following characteristics [22]:

“A total of 61 participants were enrolled, of whom 3 were excluded based on actigraphy, and 2 did not complete the study beyond the baseline DLMO. Overall, 56 healthy young Caucasian adults (29 women, 27 men; 20.8 ± 2.6 y of age) completed the study. Participants were free from any medical or psychological conditions, had a BMI of 18–30 kg/m^2^, and were not taking any medications at the time of the study. Participants had not recently traveled across time zones (1 mo per time zone, up to 3 mo) or engaged in shiftwork in the previous 12 mo. Women were naturally cycling (i.e., free from hormonal contraception) and had a regular menstrual cycle of 21–36 d in duration. Participants were healthy sleepers, reporting no subjective problems or previous diagnoses, and having a regular bedtime before 1 AM. A score of 10 or greater on the Epworth Sleepiness Scale was exclusionary, and participants were predominantly intermediate chronotypes (MEQ score of 52.7 ± 9.2). Participants had an average bedtime and waketime of 23:04 (SD = 44 min) and 07:04 (SD = 44 min), respectively. DLMO occurred on average at 21:05 (SD = 70 min), 2.22 h before bedtime. A total of nine participants wore prescription glasses at each test session.”

### 5.2. Reproducibility of Results

The version of melluxdrc used to generate the results in this paper is v1.0.0.

### 5.3. Shiny App

**Figure 9:**
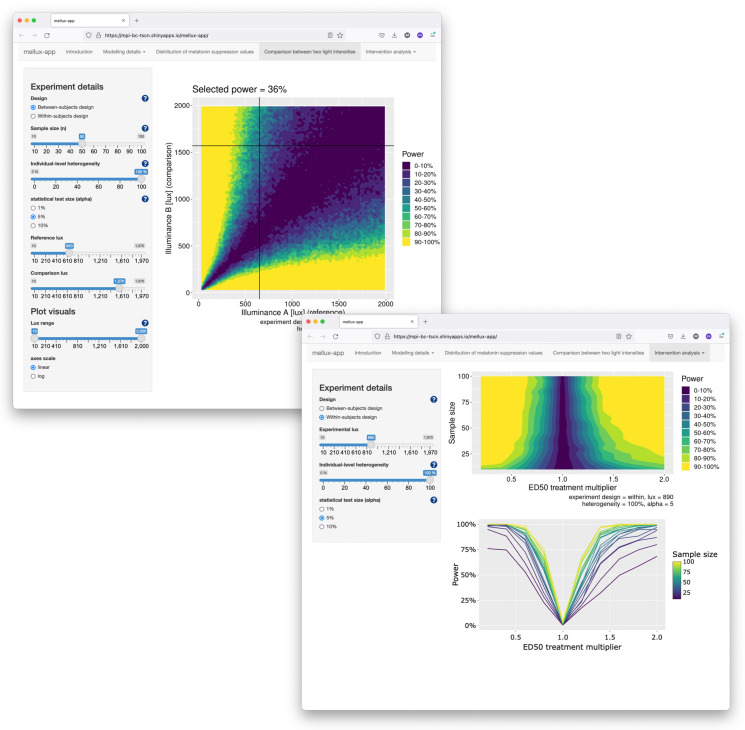
Screenshots of the Shiny app for exploring sample size calculations interactively.

We provide an app developed in the Shiny framework which allows users to generate power analyses. The app is deployed at https://tscnlab.shinyapps.io/mellux-app/ (accessed on 1 February 2024). Screenshots are shown in Figure 9.

### 5.4. Code and Data Availability

All code and data underlying this work is available under the MIT License. We make available the R package melluxdrc for generating virtual experiments [23], code to run simulations on a cluster [33], and code to fit dose response curves [29]. The code underlying the shiny app, deployed at https://tscnlab.shinyapps.io/mellux-app/ (accessed on 1 February 2024) is available as well [24].

## Figures and Tables

**Table 1:** **Priors.** Shows the priors used on the parameters of the Bayesian models that were estimated.

Model	Prior
Equation (1)	α∼normal(0, 1)
Equation (1)	β∼normal(0, 1)
Equation (1)	$\sigma_{0}∼{\mathrm{Cauchy}}^{+}\left(0,\;1\right)$
Equation (1)	$\sigma_{1}∼{\mathrm{Cauchy}}^{+}\left(0,\;1\right)$
Equation (4)	a∼Cauchy(0, 1)
Equation (4)	b∼Cauchy(0, 1)

## Data Availability

See Section 5.4.
